# Extracellular Vesicles in Alzheimer’s Disease: Dual Roles in Pathogenesis, Promising Avenues for Diagnosis and Therapy

**DOI:** 10.3390/pharmaceutics18010070

**Published:** 2026-01-05

**Authors:** Feng Li, Liyang Wu, Xin Feng, Yihong Li, Huadong Fan

**Affiliations:** 1 College of Biological & Environmental Sciences, Zhejiang Wanli University, Ningbo 315000, China; 2023881027@zwu.edu.cn; 2 Innovation Center for Diagnosis and Treatment of Neurological Disease, Ningbo Institute of Life and Health Industry, University of Chinese Academy of Sciences, Ningbo 315000, China; liyangw214@gmail.com (L.W.); fengxin10240@gmail.com (X.F.); 3 Lab of Nanopharmacology Research for Neurodegeneration, Department of Research and Development of Science and Technology, Ningbo Institute of Life and Health Industry, University of Chinese Academy of Sciences, Ningbo 315000, China; 4 Lab of Dementia and Neurorehabilitation Research, Department of Research Development of Science and Technology, Ningbo Institute of Life and Health Industry, University of Chinese Academy of Sciences, Ningbo 315000, China

**Keywords:** Alzheimer’s disease, extracellular vesicles, folate delivery, focused ultrasound, microfluidic platform

## Abstract

Alzheimer’s disease (AD) is a progressive neurodegenerative disorder characterized by the accumulation of amyloid-β (Aβ) plaques, neurofibrillary tau tangles, chronic neuroinflammation, and synaptic loss, leading to cognitive decline. Extracellular vesicles (EVs)—lipid bilayer nanoparticles secreted by nearly all cell types—have emerged as critical mediators of intercellular communication, playing a complex dual role in both the pathogenesis and potential treatment of AD. This review generally delineates two opposite roles of EVs in pathogenesis and potential treatment of AD. On one hand, EVs derived from neurons, astrocytes, microglia and oligodendrocytes can propagate toxic proteins (Aβ, tau) and inflammatory signals, thereby accelerating disease progression. On the other hand, EVs—especially those from mesenchymal stem cells (MSCs)—exert neuroprotective effects by facilitating toxic protein clearance, modulating immune responses, preserving synaptic integrity, and alleviating oxidative stress. The cargo-carrying function of EVs gives them considerable diagnostic value. The associated cargos such as proteins and microRNAs (miRNAs) in the EVs may serve as minimally invasive biomarkers for early detection and monitoring of AD. Therapeutically, engineered EVs, including those incorporating CRISPR/Cas9-based genetic modification, are being developed as sophisticated delivery platforms for targeting core AD pathologies. Furthermore, this review highlights emerging technologies such as microfluidic chips and focused ultrasound (FUS), discussing their potential to enhance the translational prospects of EV-based early diagnostic and treatment for AD.

## 1. Introduction

Alzheimer’s disease (AD) is the most common cause of dementia, affecting over 55 million people worldwide. The number is expected to rise to nearly 152.8 million by 2050, with the most significant increase anticipated in developing countries [1,2]. Pathologically, AD is characterized by the accumulation of misfolded proteins, including amyloid-β (Aβ) plaques and hyperphosphorylated tau tangles, accompanied by neuroinflammation, synaptic dysfunction, and metabolic disturbances [3]. These interconnected processes ultimately lead to progressive neuronal loss and cognitive decline [4,5]. Despite decades of intensive research, effective therapies remain elusive, as most treatments targeting single pathological factors have yielded limited clinical benefits [6].

The pathology of AD is characterized by three main features: (1) The accumulation of Aβ, considered a primary pathogenic driver [7]. Aβ is generated from the amyloid precursor protein (APP) through cleavage by β-secretase (BACE-1) and γ-secretase. The resulting Aβ monomers then aggregate into soluble oligomers, protofibrils and fibrils, ultimately forming amyloid plaques in the brain parenchyma and cerebral blood vessels [8,9]. (2) Neurofibrillary tangles (NFTs), another pathological hallmark, arise from hyper-phosphorylated and misfolded Tau proteins [3,7]. This abnormal phosphorylation disrupts tau’s association with microtubes, causing it to aggregate into NFTs within neurons and impair axonal transport and synaptic function [3]. (3) Increasing evidence identifies neuroinflammation as a critical driver and amplifier of AD pathology [10]. Activated microglia, often clustered around amyloid plaques, respond to pathogenic signals such as Aβ oligomers and other damage-associated molecules. This activation triggers the release of pro-inflammatory cytokines (e.g., IL-1β, TNF-α), chemokines, and reactive oxygen species (ROS), which further exacerbate neuronal dysfunction and synaptic damage [11]. While initially protective, sustained inflammatory responses contributes to neuronal degeneration, mitochondrial dysfunction, synaptic loss and further propagation of both Aβ and tau pathologies [9,12]. Crucially, these pathogenic factors interact synergistically to promote AD pathology. For instance, Aβ oligomers can accelerate tau hyperphosphorylation, while tau pathology facilitates the spread of neurodegeneration across the brain region [13,14].

Given their roles in mediating complex interplay among neurons, glia, and the extracellular environment, extracellular vesicles (EVs) are increasingly recognized as key participants in AD processes, facilitating both the propagation and regulation of Aβ, tau, and inflammatory signaling. EVs are nanoscale, lipid bilayer-enclosed particles secreted by nearly all cell types and are present in biological fluids [15,16,17]. Based on their biogenesis, EVs are broadly classified into exosomes, microvesicles, and apoptotic bodies [18]. Exosomes (30–150 nm) originate from the inward budding of endosomal membranes, forming multivesicular bodies (MVBs) that release intraluminal vesicles (ILVs) into the extracellular space upon fusion with the plasma membrane [19,20]. They are commonly characterized by surface markers such as CD9, CD63, CD81, ALIX (PDCD6IP), and TSG101 [21]. In contrast, microvesicles (100–1000 nm) are generated through direct outward budding and fission of the plasma membrane and are commonly enriched in CD40, selectins and integrins [22,23,24]. Apoptotic bodies (1–5 μm) are released from cells undergoing programmed cell death and typically display markers such as Annexin V [25,26]. Despite differences in size and biogenesis, all EV subtypes transport diverse molecular cargo-including proteins, lipids, nucleic acids (mRNA, miRNA), and metabolites from their parent cells—thereby facilitating intercellular communication and regulating a broad range of physiological and pathological processes [22,27,28] (Table 1).

In the context of AD, EVs exhibited a paradoxical, dual role. On one hand, they propagate disease by transferring toxic proteins such as Aβ or tau, promoting neuroinflammation and accelerating neurodegeneration [29,30]. On the other hand, EVs also exert neuroprotective effects by delivering regulatory molecules that support neuronal survival, modulate immune responses and promote repair mechanisms [31,32]. This bidirectional influence highlights their importance as both mediators and modulators of AD pathology.

Accordingly, this review provides a comprehensive overview of the multifaceted role of EVs in AD. We first discuss the classification and cellular origin of EVs subtypes involved in AD, followed by their involvement in disease pathogenesis and endogenous protective mechanisms. Subsequently, we evaluate how EVs can be harnessed for therapeutic and diagnostic applications. Finally, we explore recent advances, translational challenges, and future perspectives in leveraging EVs for the treatment and early detection of AD.

**Table 1:** The characteristics and functions of EV subtypes.

EV Subtype	Size Range	Key Surface Markers	Typical Cargoes	Primary Functions	References
Exosomes	30–150 nm	Tetraspanins (CD9, CD63, CD81), ALIX, TSG101, HSP70	Proteins, lipids, mRNAs, miRNAs, other non-coding RNAs	Intercellular signaling, immune modulation, synaptic plasticity. In AD, heavily implicated in Aβ/tau propagation and neuroinflammation.	[33,34]
Microvesicles	100–1000 nm	Integrins, selectins, CD40, ARF6, tissue factor	Cytosolic proteins, lipids, RNAs, organelles	Intercellular communication, Stimulate synaptic activity.	[24,35]
Apoptotic Bodies	1–5 μm	Phosphatidylserine (exposed), Annexin V, calreticulin, calnexin	Plasma membrane, fragmented organelles, various biomolecules (RNA and DNA)	Clearance of apoptotic debris; intercellular communication.	[21,26]
NDEVs	144–230 nm	L1CAM, GluR2/3, GAP43, NLGN3	Synaptic proteins, lipids, regulatory miRNAs (e.g., miR-21-5p), and pathogenic molecules (tau and Aβ)	Propagation of pathological proteins, participating in synaptic communication and plasticity.	[36,37,38]
ADEVs	100–160 nm	EAAT1/GLAST, GFAP	Pathogenic molecules (tau and Aβ), PAR-4 and ceramide, heat shock proteins and NTPDases	Promote neurite outgrowth, dendritic branching, neuronal survival, and synaptic plasticity, impair neuronal excitability and neurite growth.	[39,40,41]
MDEVs	98 ± 24.1 nm	CD9, CD81, CD63, MHC class II molecules	Pathogenic molecules (tau and Aβ), TRME2, Inflammatory factors (IL-1β and TNF-α), synaptogenic proteins	Propagation of Aß and Tau, release of inflammatory factors, synaptic dysfunction, alleviation of oxidative stress, neurite outgrowth.	[42,43,44]
ODEVs	30–80 nm	ALIX, TSG101, Myelin-specific proteins (PLP, CNO, MBP, MOG)	PLP, CNP, MBP, MOG, and characteristic myelin lipids	Trigger of neuronal hyperactivity, alleviation of oxidative stress synaptic preservation.	[45,46,47,48]
MSC-EVs	40–100 nm	CD9, ALIX, TSG101, CD90, CD73, and CD44	Growth factors (e.g., VEGF, TGF-β, HGF), immunomodulatory molecules (IL-10), lipids, and microRNAs (e.g., miR-21, miR-122, miR-146a)	Clearance of Aß and Tau, alleviation of oxidative stress, synaptic preservation, reduction of neuroinflammation, metabolic modulation and folate delivery.	[49,50,51,52]

## 2. Classification and Functional Heterogeneity

Beyond structural classification, the biological functions of EVs are primarily determined by the identity and physiological state of their parent cells [53]. The cellular origin governs the specific molecular composition of EV cargo, shaping their effects on recipient cells [54]. The principle is particularly significant within the complex microenvironment of the central nervous system (CNS), where the distinct cargo of EVs derived from different neural cell types—including neurons, astrocytes, microglia, oligodendrocytes, and mesenchymal stem cells—exert distinct and sometimes opposing influences on neural function and disease progression [55,56]. In AD, this cellular heterogeneity underlies the dual role of EVs: while some populations propagate pathological factors such as Aβ and tau, amplify neuroinflammation, and disseminate oxidative stress, others mediate the clearance of toxic proteins and maintain redox homeostasis, thereby protecting against neurodegeneration. Understanding this diversity is therefore crucial for deciphering disease mechanisms and harnessing EVs for therapeutic applications.

### 2.1. Neuron-Derived Extracellular Vesicles (NDEVs)

NDEVs are characterized by surface markers such as the L1 cell adhesion molecule (L1CAM), GluR2/3, GAP43 and NLGN3 [57,58]. These markers are not only enable the immunocapture and isolation of NDEVs from biofluids but also position them as promising candidates for liquid biopsy in AD [36]. The cargo of NDEVs mirrors neuronal physiology and pathology, containing a diverse array of neuron-specific molecules, includes synaptic proteins, lipids, regulatory miRNAs (e.g., miR-21-5p), and pathogenic molecules such as hyperphosphorylated tau and Aβ. Notably, although numerous studies suggest that NDEVs contribute to AD progression by facilitating the spread of Aβ and tau pathology, other reports indicate that NDEVs may also participate in synaptic maintenance and neuronal stress responses [37,59]. These seemingly conflicting observations likely depend on disease stage, neuronal metabolic status, and EV cargo composition, highlighting unresolved questions regarding the net impact of NDEVs in vivo.

### 2.2. Astrocyte-Derived Extracellular Vesicles (ADEVs)

ADEVs are identified by specific markers such as excitatory amino acid transporter 1 (EAAT1/GLAST) and glial fibrillary acidic protein (GFAP) [40,60]. In AD, their functional profile is highly context-dependent [61]. Under anti-inflammatory conditions such as ATP or IL-10 stimulation, ADEVs carry neurotropic proteins that promote neurite outgrowth, dendritic branching, neuronal survival, and synaptic plasticity [41,62,63]. In contrast, astrocytes activated by pro-inflammatory cytokines (e.g., IL-1β) release ADEVs enriched in inflammatory mediators that impair neuronal excitability and neurite growth [64,65]. This duality underscores the need for further studies to clarify how astrocyte activation states and microenvironmental cues shape EV function during different stages of AD.

### 2.3. Microglia-Derived Extracellular Vesicles (MDEVs)

MDEVs originate from the brain’s resident immune cells and are characterized by common exosomal tetraspanin markers (CD9, CD81, and CD63) [66,67]. Notably, MDEVs also express functional major histocompatibility complex (MHC) class II molecules, reflecting their immunological activity [43]. The cargo composition of MDEVs is closely linked to the activation state of their parent microglia, determining whether their function is neuroprotective or neurotoxic [43]. MDEVs derived from pro-inflammatory (M1) microglia promotes neuroinflammation and neuronal injury, while those from alternatively activated (M2) microglia carry anti-inflammatory cytokines and neuroprotective molecules that suppress inflammation and oxidative stress [67,68]. Despite substantial evidence implicating MDEVs in the amplification of neuroinflammation and tau propagation, other studies suggest that MDEVs can also facilitate debris clearance and resolution of inflammation. Such divergent findings likely reflect differences in microglial polarization states, experimental models, and disease progression, emphasizing unresolved questions regarding when MDEVs act as pathogenic mediators versus modulators of tissue repair.

### 2.4. Oligodendrocyte-Derived Extracellular Vesicles (ODEVs)

ODEVs are characterized by typical exosomal markers, such as ALIX, TSG101, as well as myelin-specific proteins. These include proteolipid protein (PLP), 2′,3′-cyclic nucleotide 3′-phosphodiesterase (CNP), myelin basic protein (MBP), myelin oligodendrocyte glycoprotein (MOG), and characteristic myelin lipids [45,46,69]. As essential mediators of oligodendrocyte-axon communication, ODEVs have been shown to be internalized by neurons via endocytosis. Functionally, they support axonal maintenance and metabolic homeostasis [47,70,71]. However, under pathological conditions, ODEVs may also contribute to disease processes, underscoring their complex and context-dependent roles in AD [47]. Notably, compared with neuronal and glial derived EVs, the involvement of ODEVs in AD remains poorly understood. Particularly, the oligodendrocytes are the primary cells for myelin sheath formation, which enables high-speed transmission of electrical signals between neurons [72]. However, whether extracellular vesicles from these cells (ODEVs) influence axonal conductance or regeneration during AD development remains unexplored.

### 2.5. Mesenchymal Stem Cell-Derived Extracellular Vesicles (MSC-EVs)

MSC-EVs are important paracrine effector molecules of mesenchymal stem cells (MSCs) and have shown therapeutic potential in diverse inflammatory and degenerative diseases [50,73]. These vesicles express universal EV markers such as CD9, ALIX, and TSG101 along with MSC-specific markers, such as CD90, CD73, and CD44 [51,74]. Their cargo includes growth factors (e.g., VEGF, TGF-β, HGF) [75,76], immunomodulatory molecules (IL-10), lipids, and microRNAs (e.g., miR-21, miR-122, miR-146a) [77,78,79]. Importantly, MSC-EVs are not a homogeneous population. Lai and colleagues were the first to demonstrate that MSCs secrete at least three distinct subtypes of EVs with differing membrane lipids, proteins and RNA cargoes, reflecting their diverse biosynthetic origins and specialized biological functions [80]. This intrinsic heterogeneity underpins their versatility as therapeutic candidates for neurodegenerative disorders, including AD.

## 3. The Dual Role of EVs in Mediating AD Pathogenesis

**Figure 1:**
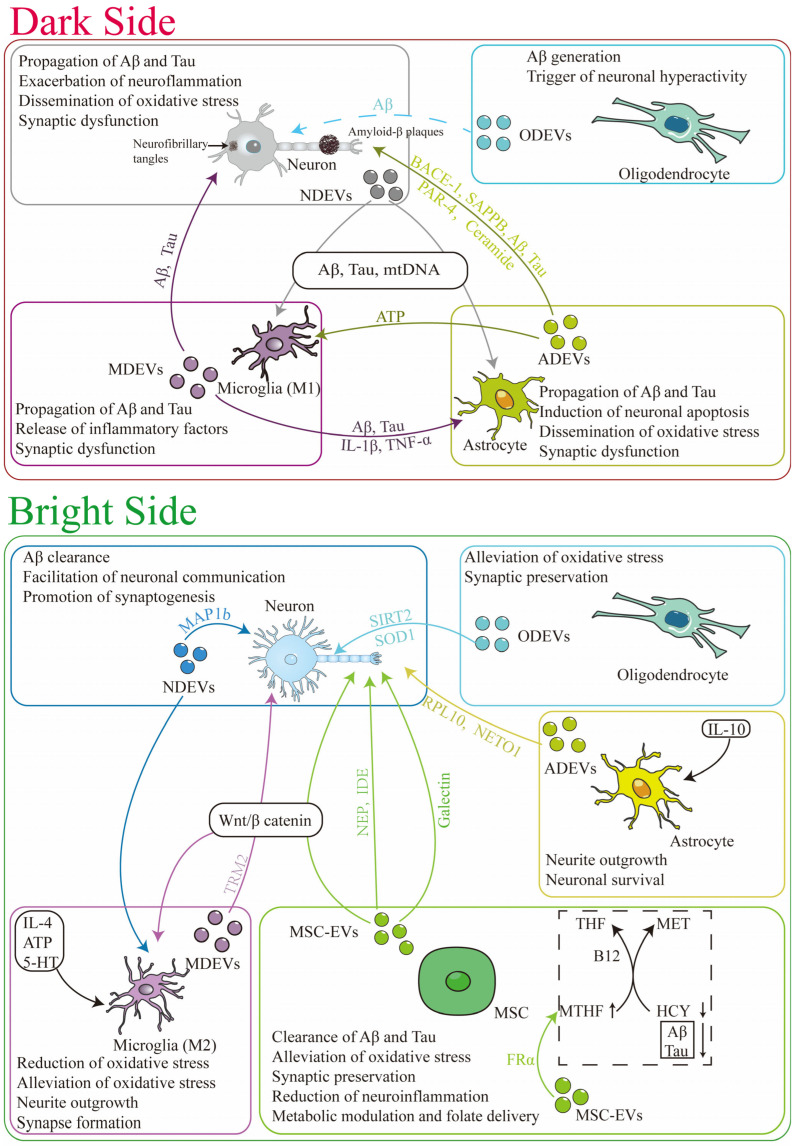
The dual role of EVs in AD pathogenesis. The schematic illustrates the opposing functions of EVs in AD, contrasting their pathological role in propagating the disease (**top**, “Dark side”) with their endogenous protective and therapeutic functions (**bottom**, “Bright side”). On the dark side, EVs derived from neurons and glial cells act as mediators of AD progression by facilitating the intercellular propagation of Aβ and tau pathologies, exacerbating neuroinflammation through the releasing of pro-inflammatory factors, and disseminating oxidative stress. Conversely, on the bright side, EVs-from endogenous neurons, glial cells and mesenchymal stem cells (MSC-EVs)-orchestrate a multifaceted defense by promoting the clearance of pathogenic Aβ and tau, delivering neuroprotective and immunomodulatory cargo to suppress inflammation, preserving synaptic integrity, alleviating oxidative stress and modulating brain metabolism.

As established in the previous section, the functional heterogeneity of EVs is intrinsically linked to the physiological state of their parent cells. This context-dependent nature determines its complex dual role in AD, wherein they can function as both drivers of pathological progression and mediators of endogenous protection. This section delineates these opposing functions, first examining the mechanisms by which EVs exacerbate core AD pathologies; namely, the propagation of Aβ and tau, the amplification of neuroinflammation, and the dissemination of oxidative stress. Subsequently, we explore the protective countermeasures orchestrated by EVs, including the clearance of toxic proteins, neuroprotection, immunomodulation, and the preservation of synaptic integrity and metabolic homeostasis (Figure 1).

### 3.1. The Dark Side: EVs as Mediators of AD Progression

#### 3.1.1. Propagation of Aβ and Tau

A key mechanism by which EVs promote AD progression involves dissemination of toxic proteins—particularly Aβ and hyperphosphorylated tau—across neuronal networks. NDEVs serve as vehicles for these pathogenic proteins [81]. They encapsulate these pathological proteins and release them into the extracellular space, these vesicles are subsequently internalized by neighboring neurons, propagating pathological tau phosphorylation and Aβ accumulation throughout interconnected brain regions [37]. Clinical evidence supports this mechanism: plasma NDEVs from AD patients contain significantly elevated levels of P-tau T181, P-tau S396, and Aβ_1–42_. Crucially, when these human-derived NDEVs are injected into mice, they induced tau pathology in the hippocampus, directly demonstrating their capacity to propagate neuropathology [38].

Beyond NDEVs, glia-derived EVs—particularly those from astrocytes and microglia—further amplify disease pathology [82,83]. ADEVs contain high levels of APP-processing components, including BACE-1, γ-secretase, soluble Aβ_42_, and soluble amyloid precursor protein β (sAPPβ), when compared with NDEVs from the same individuals [41,65]. Through this cargo, ADEVs can promote APP cleavage and intercellular transport of amyloidogenic peptides, thereby enhancing local Aβ accumulation. Furthermore, ADEVs also carry pathogenic phosphorylated tau species (p-tau T181 and p-tau S396) [84]. Similarly, MDEVs can encapsulate and release Aβ and tau, which can be transferred to neurons and other glial cells, perpetuating the pathogenic cycle [85]. Emerging evidence further implicates ODEVs may also contribute to AD pathogenesis. Oligodendrocytes have been shown to produce Aβ and to drive abnormal neuronal hyperactivity in AD model [86]. Selective suppression of oligodendrocyte-derived Aβ production rescues neuronal dysfunction and reduces plaque formation, suggesting that ODEVs may act as additional vehicle for delivering Aβ to neurons, thereby promoting neuronal dysfunction and accelerating AD pathology [87]. Collectively, these findings indicate that EVs derived from multiple neural cell types form an interconnected network that drives the intercellular spread of Aβ and tau, thereby perpetuating neurodegeneration and neuroinflammation in AD.

#### 3.1.2. Amplification of Neuroinflammation

The pathogenic role of EVs in AD extends beyond the transmission of misfolded proteins to include the modulation of neuroinflammatory signaling [88]. In this process, microglia and astrocytes, along with their secreted EVs, create a vicious inflammatory cycle that exacerbates neuronal damage. Upon pro-inflammatory activation, microglia release MDEVs loaded with cytokines such as IL-1β and TNF-α, these vesicles further propagate tau pathology by internalizing and subsequently secreting pathological tau proteins [44]. Damage-associated molecules like ATP can trigger the release of such cytokine-enriched MDEVs, which in turn intensify neuroinflammatory signaling in the brain [89,90]. Similarly, astrocytes exposed to IL-1β or Aβ release ADEVs carrying pro-inflammatory and neurotoxic cargo, including PAR-4 and ceramide, which downregulate anti-inflammatory gene expression in recipient cells and further induce neuron apoptosis [61,91]. Functionally, such ADEVs have been shown to impair neurite outgrowth, reduce neuronal excitability, and further stimulate neuronal Aβ production [61]. Together, these interactions establish a pathological feed-forward loop: neuroinflammation recruits and activates more glial cells, which in turn release more inflammatory EVs (both MDEVs and ADEVs), thereby worsening synaptic dysfunction, impairing neuronal communication, and accelerating neurodegeneration [92,93]. Furthermore, under AD-related stress, the typically homeostatic ODEVs—normally involved in maintaining myelin homeostasis—undergo composition alterations, transforming from immunologically inert vesicles into potential triggers of neuroinflammatory responses [94]. Collectively, these findings underscore that EV-mediated crosstalk among glial cells plays a central role in sustaining chronic neuroinflammation in AD.

#### 3.1.3. Dissemination of Oxidative Stress

Oxidative stress represents another key mechanism in AD pathogenesis, intricately linked with Aβ aggregation, tau pathology, and neuroinflammation [95]. Mitochondrial dysfunction is a major source of reactive oxygen species (ROS), functioning as both a cause and a consequence of oxidative damage—a hallmark of neurodegeneration [96,97,98]. Critically, this oxidative burden is not confined to individual cells but is propagated throughout the brain via EVs [99]. Under oxidative stress, neurons release EVs carrying mitochondrial DNA (mtDNA) [100]. Once released into extracellular space, these mtDNA-bearing EVs act as damage-associated molecular patterns (DAMPs) and, upon uptake by recipient microglia or astrocytes, they activate cytoplasmic DNA sensors (primarily the cGAS/STING pathway) and other pattern-recognition receptors (like endosomal TLR9), triggering a robust pro-inflammatory response that further amplifies neuroinflammation [101]. This EV-mediated interplay between oxidative damage and inflammation creates a vicious cycle that synergize with Aβ and tau pathology, ultimately driving synaptic dysfunction and neuronal loss [102]. Compounding this damage, NDEV from AD patients exhibit significantly reduced levels of key functional proteins, these include deficits in synaptic proteins such as neurogranin, synaptophysin, GAP43 [103], along with deficits in stress-response and signaling proteins, collectively impairing neuronal health [38,103,104]. The role of ADEVs in oxidative stress is complex and context-dependent. For instance, in models of amyotrophic lateral sclerosis (ALS), EVs derived from astrocytes expressing mutant SOD1 (e.g., SOD1G93A) transfer misfolded SOD1 to spinal neurons and induce selective motor neuron death, thereby exacerbating oxidative stress and neurodegeneration [64]. This paradox underscores that the functional outcome of EV-mediated transfer is critically dependent on the physiological state and cargo of the parent cell.

### 3.2. The Bright Side: Endogenous Protective Functions of EVs in AD

In contrast to their role in propagating pathology, EVs also function as vital defense system, preserving neural homeostasis and repair in AD. This section consolidates the multifaceted protective mechanisms that counter the disease processes described earlier, including clearance of toxic proteins, neuroprotection and immunomodulation, preservation of synaptic integrity, and the restoration of metabolic balance. Collectively, these functions position EVs as intrinsic defenders against the progression of AD.

#### 3.2.1. Clearance of Pathogenic Proteins

A primary protective function of EVs is the active clearance of Aβ and the reduction of pathological tau phosphorylation [105,106]. NDEVs can sequester soluble Aβ oligomers, and these EV-bound Aβ complexes are subsequently phagocytosed and degraded by microglia, thereby reducing amyloid plaque burden [107]. This endogenous clearance mechanism can be augmented therapeutically. For instance, statins promote the secretion of exosomes enriched with Insulin-degrading enzyme (IDE). These exosomes then mediate the enhanced degradation of extracellular Aβ, a process demonstrated in microglial cultures and in the circulation of mice [108]. Similarly, a single intracerebral administration of bone marrow-derived MSC-EVs was shown to reduced Aβ plaques in APP/PS1 mice, an effect potentially mediated by the presence of Aβ-degrading enzyme Neprilysin (NEP) in EVs [52]. Furthermore, BMSC-EVs containing GDF-15 upregulated the expression of IDE and NEP, thereby enhancing the clearance of Aβ_42_ and reduce its intracellular levels [109]. Our own unpublished work using broccoli-derived exosomes (BDEVs) also shows a significant reduction in hippocampus and cortical Aβ levels alongside improved cognitive function in APP/PS1 mice, underscoring the therapeutic potential of EV-mediated clearance [110].

Beyond Aβ, multiple studies have confirmed that EVs offer a potent strategy against tau pathology by targeting its hyperphosphorylation. A key mechanism involves the inhibition of glycogen synthase kinase-3β (GSK-3β) activity, a key kinase driving tau pathology [111,112]. This is evidenced by exosomes from human induced pluripotent stem cells (hiPSC-NSCs) reduced tau phosphorylation in Aβ-exposed neurons by regulating autophagy and GSK-3β activity [113]. Another example is provided by exosome derived from human umbilical cord blood-derived mesenchymal stem cells (hUCB-MSCs), which carry galectin (GAL-3), this cargo suppresses GSK-3β activity, thereby alleviating tau pathology and promote neuronal survival [114]. Collectively, these findings underscore the therapeutic promise of harnessing EVs’ native protective functions against AD.

#### 3.2.2. Neuroprotection and Immunomodulation

EVs exert direct neuroprotective and immunoregulatory effects, primarily by delivering specific cargo that counteracts neurodegeneration and inflammation [115,116]. For instance, MSC-EVs can deliver miRNA, such as miR-29c-3p, which suppresses BACE1 expression and activate the pro-survival Wnt/β-catenin signaling pathway, thereby protecting neurons from Aβ-induced oxidative stress and synaptic damage [117]. In parallel, exosomes from human umbilical cord mesenchymal stem cells (hUC-MSCs-EVs) intrinsically modulate neuroinflammation by shifting the cytokine balance toward an anti-inflammatory state [118,119]. The therapeutic potential of this immunomodulation is further demonstrated by studies showing that intranasal administration of MSC-derived exosomes significantly reduces hippocampus inflammation, enhances neurogenesis in the subventricular zone (SVZ), and lead to cognitive recovery in AD models [120].

Glia-derived EVs play a pivotal role in re-establishing immune homeostasis in the AD brain. When microglia are polarized toward a neuroprotective phenotype (M2) by signals such as interleukin (IL-4), the resulting MDEVs carry TRME2, which activating the Wnt/β-catenin signaling pathway, thereby ameliorating neuronal ferroptosis, oxidative stress and neuroinflammation in AD mouse model [67]. Similarly, ADEVs produced under anti-inflammatory conditions (e.g., IL-10 stimulation) promote neurite outgrowth and neuronal survival [41]. Furthermore, this restorative glia-immune axis can be therapeutically harnessed. For instance, hUC-MSCs-EVs can shift microglia toward an anti-inflammatory state, further enhancing Aβ clearance and reducing neuroinflammation [118]. Together, these findings indicate that endogenous EVs function as physiological messengers of neuroprotection, capable of resetting dysregulated inflammatory networks in AD.

#### 3.2.3. Synaptic Preservation

Synaptic dysfunction and loss is a strong correlate of cognitive decline in AD, and EVs play a direct role in preserving synaptic integrity [121]. In vitro studies using microfluidic chambers have shown that NDEVs are internalized by recipient neurons and can undergo transsynaptic transfer, facilitating inter-neuronal communication and promoting excitatory synaptogenesis [122]. Supporting this concept, exosome derived from neuronal cells or human cerebrospinal fluid can neutralize the synaptotoxic effects of Aβ oligomers, preserving synaptic plasticity and preventing cognitive impairment in AD models [123]. The release and molecular composition of neuronal EVs are closely coupled to neuronal activity. For example, depolarization-induced NDEVs that are enriched with microtubule-associated protein 1b (MAP1b) could further enhance synaptic plasticity and connectivity [124]. Furthermore, glia-derived EVs provide essential support to this neuronal network. ODEVs are critical for maintaining axonal integrity and neuronal health [71]. Notably, ODEVs deliver SIRT2 protein, which has been shown to restore hippocampal neurogenesis and synaptic plasticity through activation of the AKT/GSK-3β signaling pathway [125]. Similarly, MDEVs also contribute to synaptic maintenance in a state-dependent manner, with their release and cargo composition being directly regulated by neuronal activity [126]. This secretion can be influenced by specific signals, including neurotransmitter serotonin (5-HT) and the Wnt3a pathway. Notably, ATP stimulation triggers the secretion of MDEVs enriched with synaptogenic proteins like thrombospondin-1 and thrombospondin-4, which actively promote neurite outgrowth and synapse formation [126,127,128]. Collectively, EVs from neurons and glia form a coordinated communication system that is vital for preserving synaptic integrity in the face of AD pathology.

#### 3.2.4. Alleviation of Oxidative Stress

Oxidative stress, characterized by a persistent imbalance between ROS production and cellular antioxidant capacity, is a critical driver of pathogenesis of AD [129]. Within this context, EVs—particularly those from microglia and oligodendrocytes—have emerged as key regulators of the cerebral redox environment [130,131]. A pivotal mechanism involves the packaging and intercellular delivery of antioxidant enzymes. For instance, Peng et al. demonstrated that MDEVs could mitigate oxidative stress and promote angiogenesis by activating the Keap1/Nrf2/HO-1 signaling pathway, upregulating the antioxidant genes like HO-1, NQO1, Gclc, Cat, and Gsxl [131]. Alongside MDEVs, ODEVs also play an essential role in maintaining redox homeostasis. ODEVs facilitate the intercellular transfer of powerful antioxidant enzymes, including SOD1 and catalase, establishing an essential oligodendrocyte–neuron axis that helps maintain redox balance in the CNS [48]. Furthermore, ADEVs contribute to protection by carrying stress-responsive cargo. This includes heat shock proteins and nucleoside triphosphate diphosphohydrolases (NTPDases), which help mitigate metabolic or thermal stress and support neuronal survival [61,132]. Together, this EV-mediated crosstalk constitutes a fundamental, multi-cellular strategy for the CNS to mitigate oxidative stress and maintain neuronal homeostasis.

#### 3.2.5. Metabolic Modulation and Folate Delivery

Emerging evidence links metabolic disturbances to AD pathogenesis; particularly hyperhomocysteinemia (HHcy), which is widely recognized as a significant risk factor for cognitive decline and AD [133]. A meta-analysis of 35 studies confirmed that blood homocysteine (Hcy) levels in AD patients are approximately one-third higher than in healthy controls [134]. The pathogenic mechanism underlying HHcy in AD involves in the promotion of oxidative stress and neuroinflammation, which directly exacerbate Aβ deposition and tau hyperphosphorylation [135,136,137]. At molecular level, HHcy increases the generation of Hcy-thiolactone (HTL), leading to aberrant protein N-homocysteinylation that disrupts protein structure and induces neurotoxicity [137]. Furthermore, HHcy can drive AD pathology by upregulating the mTOR signaling pathway, thereby inhibiting autophagy and reducing the clearance of Aβ [138]. Notably, HHcy often presents alongside deficiencies in folate and vitamin B12, and this combination exhibits a strong association with AD [139]. This suggests that correcting these nutritional deficiencies could be a viable therapeutic strategy to lower Hcy levels and potentially show disease progression. In addition, epidemiological evidence further support this, indicating that high folate intake is associated with a reduced risk of AD [140]. However, the efficiency of dietary folate is limited by its inefficient delivery to the brain parenchyma.

We hypothesize that the efficient delivery of folate to the brain parenchymal largely depend on the specific recognition of folate-receptor α (FRα), which is highly expressed in MSC-exo, results in efficient delivery of folate reach the neurons or glia. Supporting this hypothesis, recent data in our lab has shown that intranasal administration of low-dose MSC-exo significantly alleviates cognitive deficits and reduce Aβ accumulation in AD mouse model. Crucially, this treatment led to a significant increase in brain 5-Methyltetrahydrofolic acid (5-MTHF) and a concurrent decrease in HCY levels [141]. This preliminary data indicates a promising mechanism whereby MSC-EVs, via their surface FRα, could enhance folate uptake in the brain parenchyma and ultimately counteracting HHcy in AD.

The dual role of EVs in AD is governed by specific contextual factors that determine their function output. (1) Local tissue conditions critically influence EV biogenesis and release: pro-inflammatory signal (TNFα, IL-1β, Aβ) promote the secretion of ADEVs carrying DAMPs, cytokines, and pathogenic proteins, thereby promoting neuroinflammation and oxidative stress [61,91]. Conversely, anti-inflammatory molecules (IL-4, IL-10) or specific neuro-modulatory signals (e.g., 5-HT) favor the release of MDEVs loaded with growth factors, antioxidant enzymes, and miRNA that suppress inflammation and support neuronal repair [126,127]. Metabolic stressors such as HHcy or metabolic dysfunction can also alter EV cargo, lining systemic metabolic disturbances to CNS EV signaling. (2) These extracellular cues ultimately influence parent cell’s identity and physiological state, which directly dictate EV cargo composition and function. For example, MDEVs can be either neurotoxic (when derived from pro-inflammatory M1 microglia) or neuroprotective (from anti-inflammatory M2 phenotype), depending on local cytokines such as damage-associated molecules ATP or IL-4 [67]. Similarly, ADEVs could shift from promoting neurite outgrowth under anti-inflammatory conditions (e.g., IL-10 stimulation) to impairing neuronal function under pro-inflammatory stimuli (e.g., IL-1β or Aβ exposure) [41,126]. (3) Furthermore, the net functional impact of EVs may evolve with AD progression. In the initial stages, EVs may participate in compensatory clearance of Aβ and tau, support synaptic plasticity, and modulate immune surveillance [106,107,117,122]. As pathology progressed, chronic stress and inflammation drive the release of EVs enriched with pathogenic proteins (Aβ, tau) and inflammatory factor, accelerating disease spread and neuronal damage [81,82].

Together, these findings establish EVs as intrinsic guardians of neural homeostasis. Their ability to clear pathogenic proteins, deliver neuroprotective cargo and coordinate intercellular defense mechanisms underpins their promise as naturally evolved therapeutic vehicles.

## 4. Harnessing EVs for Therapy: Targeting Core AD Pathologies

The complex pathology of AD presents multiple therapeutic targets, with the aggregation of Aβ, hyperphosphorylation of tau, and chronic neuroinflammation being the most prominent. EVs, particularly when engineered, offer innovative and targeted strategies to address these core pathologies. This section explores how EV-based approaches are designed to directly target the key mechanisms driving AD progression.

### 4.1. Engineered EVs for Targeting Aβ and Tau Pathology

The propagation and accumulation of Aβ and hyperphosphorylated tau are central to AD pathogenesis. EVs are being engineered to enhance the degradation and clearance of these toxic proteins, moving beyond their natural capabilities. For example, to boost Aβ clearance, researchers have developed mannose-modified exosomes loaded with gemfibrozil (MExo-Gem), which specifically target microglia. This strategy enhances their lysosomal activity, leading to reduced amyloid burden and improved cognitive function in AD mice [142]. Beyond Aβ, EVs are also being harnessed to target tau pathology. One study used EVs derived from curcumin-primed macrophages (Exo-cur), which delivered curcumin to the brain and inhibited tau protein hyperphosphorylation via the AKT/GSK-3β pathway [112]. Furthermore, an approach used NDEV engineered to overexpress Fe65, enabling them to deliver the autophagy-inducer Corynoxine-B specifically to APP-expressing neurons. This strategy not only promoted Aβ clearance via autophagy but also ameliorated cognitive deficits by enhancing synaptic regeneration in AD mouse model [143]. These strategies expand upon EV’s natural capacity for Aβ and tau pathology, converting them into programmed nanocarriers for Aβ and tau-targeted therapy.

### 4.2. Suppressing Neuroinflammation with Engineered EVs

Chronic neuroinflammation, driven by activated microglia and astrocytes, significantly contributes to AD progression. Engineered EVs, particularly those derived from Mesenchymal stem cells (MSC-EVs), have emerged as potent anti-inflammatory delivery systems. For example, human umbilical cord MSC-derived EVs loaded with miR-146a-5p effectively suppress the IRAK1/TRAF6-NF-kB signaling cascade, thereby reducing the production of pro-inflammatory cytokines such as IL-6 and TNFα [144]. Likewise, MSC-derived exosomes engineered with the brain-targeting RVG peptide (MSC-RVG-Exo) attenuate neuroinflammation in AD mouse models by rebalancing the brain’s cytokine profile, suppressing pro-inflammatory factors while promoting anti-inflammatory ones [145]. Moreover, exosomes from MSC preconditioned by hypoxia further ameliorate neuroinflammation by downregulating TNF-α, IL-1β and upregulating of IL-4, IL-10 [146,147]. These immunomodulatory strategies highlight the remarkable versatility of engineered EVs in reprogramming the neuroimmune microenvironment toward a protective, anti-inflammatory state.

### 4.3. Advanced Engineering: EVs as Delivery Systems for Genetic and Epigenetic Therapy

A next-generation strategy for AD therapy involves using EVs as delivery vehicles for precise genetic interventions, such as the CRISPR/Cas9 systems. This technology holds remarkable potential for targeting genes implicated in AD pathogenesis, such as knocking out *MAPT* (Tau) to prevent neurofibrillary tangle formation and disrupting GSAP to modulate γ-secretase activity [148]. Moreover, this approach has been used to target AD by directly correcting disease-causing mutations in genes like APP, PSEN1 and PSEN2 to reduce the production of toxic Aβ [148,149,150]. The pioneering work of Alvare-Erviti et al. demonstrated this potential by using engineered exosomes fusing with rabies viral glycoprotein (RVG) peptides to enable specific brain targeting. Injection of these RVG modified exosomes carrying BACE-specific siRNA led to a significant knockdown of BACE1 in the mouse brain [151]. Building on these advances, Han et al. developed a novel photoinducible exosome system (MAPLEX) capable of delivering functional proteins, including a Cas9-DNMT3A epigenome-editing complex targeting *BACE1* gene. This approach successfully reduced BACE1 expression, ameliorating memory deficits and decreasing amyloid pathology [152]. Importantly, this study demonstrated that exosomes can safely and efficiently deliver the CRISPR-Cas9 system to target cells while minimizing off-target effects in surrounding tissues [153]. Although CRISPR-Cas9 based EV therapies are still in early developmental stage, continuous optimization of EV loading efficiency and targeting specificity is paving the way for CRISPR-edited EVs to emerge as a next generation precision medicine tool for directly modifying core AD pathologies.

## 5. EVs as a Diagnostic Tool

Biomarkers are crucial for AD, as neuropathological changes begin years before clinical symptoms appear. Therefore, early diagnosis is crucial for intervention of AD progress. Based on the 2018 National Institute on Aging-Alzheimer’s association (NIA-AA) research framework, defines AD biologically based on three core pathological processes—amyloid beta (A), tau (T) and neurodegeneration (N)—collectively termed the ATN framework [154]. However, this framework currently relies on established cerebrospinal fluid (CSF) analysis and neuroimaging techniques like positron emission tomography (PET) and magnetic resonance imaging (MRI). While informative, these methods are limited by high costs, invasiveness, and limited accessibility, restricting their use for widespread screening and longitudinal monitoring. Consequently, the development of blood-based biomarkers has become a major focus. The plasma Aβ_42/40_ ratio [155,156,157,158], and phosphorylated tau (p-tau) species, including p-tau181, p-tau217, and p-tau 231 [159,160,161,162,163,164] show promise as A and T biomarkers, respectively, in the AT(N) framework [165,166]. A study identified plasma brain-derived tau (BD-tau) as a novel blood-based biomarker that is strongly correlated with CSF BD-tau, accurately distinguishes autopsy-confirmed AD from other neurodegenerative disease, and associated with Aβ and neurofibrillary tangle [167]. Using the Quanterix Simoa quantification system, a recent Swedish population-based cohort study followed 2148 dementia-free older adults for up to 16 years and found blood level of the biomarkers p-tau181, p-tau217, NFL, and GFP were significantly associated with an increased risk of developing all-cause and Alzheimer’s dementia [168].

Even though blood-based Aβ and p-tau show great potential for diagnosing AD, they present several limitations. The sensitivity of assays measuring blood-based Aβ can be impacted by peripheral sources, and the magnitude of change detected in blood is often less pronounced than in CSF [169,170]. Furthermore, many biomarker-detection methods can be relatively costly for specific patient demographics. Critically, current blood-based biomarkers fail to provide region-specific information about brain pathology, limiting their utility for disease staging and tracking progression [171].

In this context, EVs, particularly those derived from the CNS, have emerged as a revolutionary diagnostic platform. Their unique biological properties—including the ability to cross blood–brain barrier, low immunogenicity, and capacity to carry disease-related biomolecules from their parent cells—make them ideal “liquid biopsies” of the brain [172,173]. The analysis of EV cargo in biofluids like blood or CSF can reveal host cell biological changes, predict dementia onset, reflect cognitive changes, track treatment responses, and evaluate brain recovery [37].

### 5.1. Neuron-Derived EVs: Capturing Core Pathology and Synaptic Integrity

NDEVs hold significant promises as diagnostic biomarkers because their cargo directly mirrors the physiological and pathological state of neurons. Studies have demonstrated that the levels of pathogenic proteins in NDEVs isolated from peripheral blood strongly correlate with those in CSF and exhibit high diagnostic accuracy for AD [174]. Furthermore, NDEVs levels of Aβ_42_ increase progressively across the AD continuum—from cognitively normal amyloid-negative individuals to amyloid-positive individuals, to those with mild cognitive impairment (MCI), and finally to AD dementia—and correlate with cerebral amyloid burden on PET imaging, predicting future cognitive decline and entorhinal cortex atrophy [175]. The predictive power of NDEVs is further highlighted by studies showing that a model incorporating p-T181-tau, p-S396-tau, and Aβ_1–42_ from plasma NDEVs could correctly classified 96.4% of AD patients and predict disease onset up to 10 years before clinical diagnosis [176]. Similarly, abnormal levels of P-tau, Aβ_1–42_, neurogranin (NRGN), and repressor element 1-silencing transcriptional factor (REST) in plasma NDEVs can accurately predict conversion from MCI to AD dementia [38].

Beyond core pathologies, NDEVs also provide a window into synaptic integrity, the loss of which is a strong correlation of cognitive decline [177]. Synaptic proteins such as NRGN, synaptophysin, synaptopodin within NDEVs are progressively lower in patients with MCI and AD compared to cognitively normal controls (CNC) [38,177]. A more detailed analysis measuring five synaptic proteins in NDEVs (syntaxin 1, GAP43, GluR2, PSD95, and NRGN) revealed a complex signature: while most proteins were reduced, NRGN levels were elevated in early AD patients. A discriminant model incorporating GluR2, proBDNF, NRGN and GAP43 achieved an AD classification accuracy of 81.3%. Crucially, levels of these synaptic proteins correlated significantly with cognitive scores (MMSE and COR-SOB), suggesting they are not only for diagnosis but also for monitoring disease progression [178].

### 5.2. The Diagnostic Potential of miRNA and Glial Cells-Derived EVs

The diagnostic potential of EVs extends beyond proteins to their nucleic acid cargo. miRNAs within EVs are also potential biomarker candidates due to their stability in extracellular circulation and their involvement in regulating key biological processes in both pathological and physiological state, including neuronal proliferation and development [179,180]. In the AD brain, specific miRNA are dysregulated, including the downregulation of miRNAs that regulate BACE1 and APP (e.g., miR-29a/b-1, miR-107), and the upregulation of those that enhance tau phosphorylation and neuroinflammation (e.g., miR-125b, miR-146a) [181]. Recent reviews have identified 13 upregulated miRNAs and 14 downregulated miRNAs associated with AD pathology [182]. Detecting these dysregulated miRNAs in readily accessible biofluids like plasma and CSF positions EV-associated miRNA as promising, minimally invasive biomarkers for the early diagnosis and tracking of AD.

EV derived from other CNS cell types also offer unique diagnostic insights. The contents of ADEVs vary with disease state, and their associated pathological factors can serve as biomarkers for CNS diseases [84]. For instance, ADEVs from AD patients contain significantly elevated levels of proteins directly involved in Aβ pathology, such as BACE-1 and aAPPβ, which can effectively distinguish AD patients from healthy controls with high accuracy (AUCs of 0.78 and 0.83, respectively) [65]. Furthermore, the complement protein profile within ADEVs undergoes significant alterations during the progression from MCI to AD. Specifically, patients with MCI who converted to AD dementia (MCIC) showed significantly elevated levels of classical and alternative pathway effector proteins—including C1q, C4b, factor D, Bb, C3b, C5b, and the C5b-C9 terminal complement complex—compared to those with stable MCI (MCIS) [183]. These findings not only differentiate AD patients from controls but also exhibit high diagnostic sensitivity for identifying MCI patients at high risk of converting to AD (Table 2).

## 6. Promising Therapeutic Strategy: Link to Clinical Treatment

The preceding sections have detailed the functional heterogeneity of EVs, underscoring that the role of endogenous EVs in AD is complex and context-dependent. As demonstrated, EVs derived from neurons and glial cells (NDEVs, ADEVs, MDEVs, ODEVs) can paradoxically both propagate pathology and mediate protection, with their function determined by the physiological state of their parent cells. By harnessing EVs from beneficial sources or engineering them to override pathological cues, their innate biological properties can be leveraged for intervention. In contrast to these native vesicles, MSC-EVs have emerged as exogenous therapeutic candidates. They have emerged as particularly promising therapeutic vectors due to their consistently demonstrated protective and regenerative functions in preclinical models. In this section, we outline the key inherent advantages of EVs that position them as next-generation therapeutics for AD.

### 6.1. Biocompatibility and Low Immunogenicity

EVs exhibit exceptional biocompatibility and a favorable safety profile, primarily due to their endogenous origin, which minimizes immunological reactivity and makes them better tolerated than synthetic nanoparticles [191,192,193,194]. As naturally derived from host cells, exosomes inherently possess low immunogenicity [192]. Studies have shown that MSC-derived EVs maintain low immunogenicity even after repeated administrations, without triggering significant inflammatory responses [195]. Critically, this safety extends to engineered EVs; for instance, systemic administration of exosomes modified with anchor peptides and splicing-switching oligonucleotides result in no detectable toxicity or inflammation in major organs like the liver, kidney, and muscles [196]. This high degree of tolerability is particularly crucial for chronic neurodegenerative diseases like AD, where long-term treatment is required [197]. Furthermore, as non-replicative and non-mutagenic entities, EVs avoids regulatory concerns associated with adverse effects or tumorigenesis [198]. Collectively, their high biocompatibility and established safety profile not only enhances patient tolerance but also provides a strong foundation for the broad application of EVs in regenerative medicine.

### 6.2. BBB Penetration

The ability of EVs to cross the highly selectively BBB represents a significant therapeutic advantage for treating AD, where the efficient delivery of therapeutic to the brain remains a major obstacle [199]. Owing to their nanoscale size (30–150 nm) and endogenous lipid bilayer membrane, EVs can traverse the BBB through multiple mechanisms, including transcytosis (such as clathrin-mediated, caveola-mediated, and adsorptive-mediated endocytosis), as well as via paracellular pathway when the BBB compromised under pathological conditions [198]. This membrane composition not only protects encapsulated cargo from degradation but also facilitates its stable delivery to brain tissues [200,201]. Preclinical studies have confirmed that intravenously or intranasally administered MSC-EVs accumulate in the brain parenchyma [202,203], where they deliver functional miRNAs and growth factors that alleviate neurodegeneration and promote neurogenesis [203]. Supporting this, our preliminary data indicate that intranasal delivery of MSC-EVs effectively penetrates the BBB and alleviates AD pathology [141]. This intrinsic BBB-penetrating capability confers a clear edge over conventional macromolecules and antibodies, which often fail to reach effective concentrations in the brain, underscoring the inherent advantage of MSC-EVs for treating neurodegenerative disease like AD.

### 6.3. Regenerative Potential via Wnt/β-Catenin Signaling

Extensive studies have identified dysregulation of Wnt signaling as a contributing factor in AD pathogenesis. For instance, a recent study highlighted that downregulation of Wnt1, PORCN, and RSPO2 in the brains of AD patients inhibits the Wnt/β-catenin signaling pathway [204]. Given its crucial role in AD, Wnt signaling represents a compelling target for therapeutic intervention [205,206,207,208,209]. Beyond functioning as delivery vehicles, MSC-EVs exert regenerative effects by modulating key signaling pathways, particularly Wnt/β-catenin [210]. A key study by Sha et al. provides a direct mechanistic link, demonstrating that BM-MSC-EVs carry and deliver microRNA-29c-3p to neuronal cells. This miRNA subsequently inhibits BACE1, which in turn leads to the activation of the Wnt/β-catenin pathway, ameliorating AD pathology in rat models [117]. This pathway plays a central role in regulating neuronal proliferation, differentiation, survival, and BBB repair, making it a critical mediator of neurogenesis and angiogenesis—processes which are essential for repairing AD-damaged neural circuits [211]. By delivering specific miRNAs and growth factors, MSC-EVs activate the Wnt/β-catenin pathway in recipient cells, leading to the stabilization and nuclear translocation of β-catenin. This, in turn, orchestrates the transcription of genes involved in synaptic plasticity, neuronal survival, and the formation of new blood vessels [212,213]. For instance, adipose-derived MSC exosomes promote angiogenesis in endothelial cells by activating this pathway [214]. Similarly, MSC-EVs support bone regeneration by reducing oxidative stress and restoring cellular proliferation [215], and enhance follicular regeneration through pathway activation [216]. The activation of the Wnt/β-catenin pathway serves as a unifying mechanism, stabilizing β-catenin to coordinate the complex cellular interactions required for effective neural repair. Through enhancing neurogenesis and angiogenesis, MSC-EVs represent an innovative therapeutic strategy for AD by restoring neural integrity and function mediated by Wnt signaling.

### 6.4. Clinical Translation: Ongoing Trails and Early Outcomes

The therapeutic potential of MSC-EVs, as detailed in the preceding sections, is increasingly being validated in clinical settings. The intrinsic advantages position them as promising candidates for clinical translation, particularly for complex neurodegenerative disorders like AD where treatment options remain limited.

Pioneering clinical trials are now evaluating the safety and efficacy of EV-based therapies across a range of diseases. A notable Phase I//II trial (NCT04388982) is assessing the safety and efficacy of intranasally administered allogeneic adipose-derived MSC-EVs (ahaMSCs-Exos) in patients with mild to moderate AD. Participants received twice-weekly administrations over 12 weeks. Preliminary reports indicate that the treatment was well-tolerated across all dosage groups and was associated with improvements in cognitive function. Notably, the medium-dose group exhibited reduced hippocampal atrophy, suggesting a potential neuroprotective effect. While significant changes in amyloid or tau deposition were not observed across the cohorts, these early outcomes are promising, demonstrating the capacity of MSC-EVs to modulate disease progression and preserve brain structure [217]. Supporting these findings, another clinical study involving 13 AD patients demonstrated that intranasal administration of MSC-EVs over 8 weeks raised no safety concerns and was associated with significant improvement in the Hasegawa Revised Dementia Scale (HDS-R) scores. Increased levels of apolipoprotein, a molecule involved in Aβ clearance, further positioning intranasal MSC-EVs as a safe and convenient therapeutic for AD [218].

Beyond AD-focused studies, other clinical trials provide corroborative evidence for the CNS applicability of EV-based therapies. A Phase I//II clinical trial for acute ischemic stroke (NCT03384433) is evaluating the safety and efficacy of bone marrow MSC-EVs transfected with miR-124. Preliminary data suggests that stereotactic administration is safe, with functional recovery outcomes still under assessment. These promising results highlight the potential of engineered EVs for targeted CNS repair.

The clinical exploration of MSC-EVs extends to other neurological and systemic conditions, reflecting their broad therapeutic utility. Active or completed trials are investigating their use in Parkinson’s disease (NCT05152394), amyotrophic lateral sclerosis (NCT07105371), and psychiatric disorders such as depression and anxiety (NCT04202770). Furthermore, their regenerative and immunomodulatory properties are being harnessed in dermatology for conditions like atopic dermatitis (NCT05969717) and chronic wounds (NCT02565264), as well as in pulmonology for COVID-19-related ARDS, showcasing a versatile and expanding clinical landscape. While larger, multi-center trails with longer follow-up periods are needed to establish their efficacy and optimal dosing regimens, the current clinical data underscore the translational premise of MSC-EVs as a next-generation, cell-free therapeutic strategy for AD.

### 6.5. Industrial Development and Commercialization of Exosome-Based Therapeutics for AD

In parallel with academic and clinical advances, increasing industrial investment underscores the translational potential of EV-based therapeutics. Several biotechnology companies and small-to-medium enterprises (SMEs), including Aegle Therapeutics [219], Codiak Bioscience [220], and Evox Therapeutics [221] are advancing exosome-based delivery systems designed to enhance tissue targeting, cargo stability, and biological compatibility [222]. In addition, companies such as Exo Biologics have established GMP-compliant manufacturing infrastructure to support the scalable production of EV-based products for clinical and potential commercial needs [223]. Although most commercial programs remain at preclinical or early clinical stages, these initiatives highlight growing confidence in EVs as next-generation delivery systems for neurodegenerative diseases, including AD.

## 7. Challenges and Limitations on the Path to Translation

Despite the considerable promise of MSC-EV therapy as a cutting-edge “cell-free” regenerative strategy, several major challenges must be addressed before its widespread clinical translation can be realized. These limitations span from production and characterization to delivery and long-term safety.

### 7.1. Heterogeneity and Standardization

A primary obstacle is the intrinsic heterogeneity of EV populations. Variations in vesicle size, cargo composition (including proteins, lipids, and nucleic acids), and functional properties are influenced by the MSC source, culture conditions, and isolation methods [224]. This biological diversity is further compounded by differences in the physiological and pathological state of the parent cells, resulting in substantial batch-to-batch inconsistency [225,226]. For example, MSCs from the same donor exhibit significant differences in composition, bioactivity, and therapeutic potential among EVs derived from different subtypes [225]. Similarly, EVs derived from other CNS cell types (e.g., astrocytes, neurons, microglia) exhibit divergent or even opposing biological functions, complicating efforts to standardize EV-based interventions [182]. Thus, robust criteria for EV identity, purity, potency and functional characterization remain urgently needed to ensure reproducibility across laboratories and clinical settings.

### 7.2. Scalable Manufacturing and GMP Compliance

Translating EV therapeutics from bench to bedside demands scalable and reproducible manufacturing pipelines. However, conventional isolation techniques, particularly ultracentrifugation, are often time-consuming, yield inconsistent purity, and are difficult to scale [227,228]. Such inconsistencies in active ingredient content between batches make it difficult to meet the stringent Good Manufacturing Practice (GMP) standards required for drug uniformity [229]. Although alternative methods such as tangential flow filtration offer improved yield, reproducibility, and scalability, further optimization is still needed for robust industrial application [230,231]. Additionally, the large-scale expansion of MSCs under GMP conditions is resource-intensive and costly, significantly limiting the broader availability and commercialization of EV-based therapeutics.

### 7.3. Targeting Specificity and Delivery Efficiency

Achieving precise targeting of EVs to specific cell types in the complex brain environment is crucial, as it maximizes therapeutic efficacy and minimizes off-target effects. The natural tropism of unmodified EVs is often non-specific, leading to widespread biodistribution and potential dilution of therapeutic impact [232]. Surface engineering approaches, such as decorating EVs membranes with targeting peptides (e.g., RVG peptides for acetylcholine receptors on neuronal cells), have improved uptake in AD-relevant regions in preclinical models [151]. However, the long-term safety, immunogenicity, and functional stability of such engineered EVs in humans still require thorough evaluation. Additionally, the presence of the BBB and the dynamic brain microenvironment further complicates efficient and sustained delivery, underscoring the need to optimize the innovation in EV modification and administration routes.

### 7.4. Long-Term Safety and Clinical Validation

Although MSC-EVs have demonstrated favorable safety profiles and significant therapeutic efficacy in animal models-such as in AD and mild cognitive impairment (MCI) models, where they effectively improved pathological indicators by modulating immune responses, promoting tissue repair, and suppressing inflammation without observed toxicity. However, their long-term safety and efficacy in human patients remain incompletely established [233]. While clinical trials have shown promising tolerability and preliminary benefits in conditions including COVID-19 [234,235] and Type 1 Diabetes (NCT02138331), most studies are still in their early stages, with limited sample sizes and short follow-up periods. Therefore, to fully validate the therapeutic potential of MSC-EVs, multi-center, large-scale, and long-term clinical trials are necessary to determine optimal dosing regimens (e.g., dose, frequency, and administration route) and establish standardized preparation protocols to ensure the quality of EVs. Concurrently, fundamental research should be strengthened to elucidate their mechanisms of action and interactions with MSC-EVs, providing a scientific basis for their safety and effective translation from the laboratory to the clinic.

## 8. Future Perspectives

**Figure 2:**
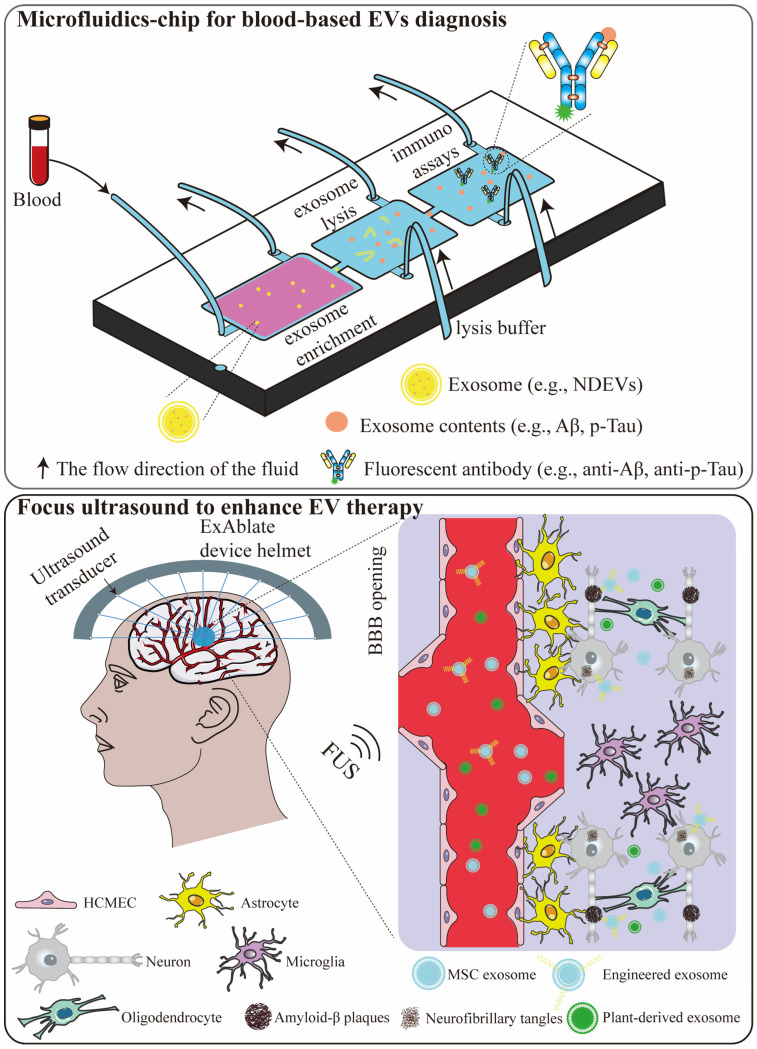
Future perspectives: Integrated EV-based platforms for AD diagnosis and therapy. Schematic overview of two promising technologies for the diagnosis and treatment of AD. Top panel: Microfluidic chip for blood-based EVs diagnostics. This platform outlines a “liquid biopsy” workflow for AD. Plasma is introduced into a microfluidic chip where NDEVs are selectively immunocaptured using antibody against neuronal surface markers (e.g., L1CAM). The captured exosomes are subsequently lysed, and their cargo (e.g., Aβ, p-tau, synaptic proteins) is quantitatively analyzed via on-chip immunoassays, enabling minimally invasive diagnosis and disease monitoring. Bottom panel: Focused Ultrasound to enhance EV therapy. A non-invasive FUS device transiently opens the BBB, intrinsically facilitates Aβ clearance, and restore cognitive function. This physical disruption enhanced the delivery of therapeutic EVs—including MSC-EVs, engineered EVs (e.g., loaded with CRISPR/Cas9 systems or anti-inflammatory miRNAs) and plant-derived EVs (e.g., broccoli-derived exosomes)—to the brain parenchyma, amplifying their therapeutic effect on core AD pathologies.

Looking ahead, several emerging technologies and integrated strategies hold significant potential to advance the use of EVs in diagnosis and treatment of AD. These approaches aim to overcome existing limitations in specificity, delivery, and therapeutic efficacy, paving the way for more precise and powerful interventions (Figure 2).

### 8.1. Microfluidic Chip for Blood-Based EVs Diagnostics

Microfluidic technology represents a promising strategy for developing blood-based diagnostic platforms for AD. Since 2010, microfluidic systems have been fabricated for exosome manipulation, offering unique advantages such as small sample volume, low cost, product purity, and short operation time [236]. For instance, a ciliated micropillar microfluidic device was designed to traps exosome-sized vesicles via size-selective filtration [237]. In one notable application, an integrated microfluidic chip was developed that combines immunoisolation with on-chip analysis of plasma-derived exosomes, establishing a promising liquid biopsy platform for non-invasive cancer diagnosis [238]. Building on this, the same research group created an ExoSearch chip for the immunomagnetic isolation and multiplexed detection of circulating exosomes, enabling blood-based diagnosis of ovarian cancer through simultaneous quantification of three exosomal tumor markers [239].

Looking forward, this lab-on-chip technology could be strategically adapted to address critical challenges in AD diagnostics. By utilizing antibodies against neuron-specific surface markers (e.g., L1CAM), such a chip could selectively enrich NDEVs from peripheral blood, serving as a real-time “liquid biopsy” of the brain. Furthermore, the integrated design would allow for the subsequent on-chip lysis of captured NDEVs and the quantitative analysis of intravascular protein targets relevant to AD pathogenesis (e.g., Aβ, p-tau, p-tau217). This microfluidic approach could thus act as a powerful and practical tool for the early detection of molecular changes in AD, potentially identifying at-risk individuals’ years before clinical onset.

### 8.2. Focused Ultrasound to Enhance EV Therapy

Focused ultrasound (FUS) has emerged as a promising non-invasive and highly controllable technique to transiently open the BBB. This capability provides a powerful means to enhance drug delivery to target brain regions in AD. Preclinical and clinical studies have demonstrated that MRI-guided FUS could safely, reversibly, and repeatedly open the BBB in AD animal model even in human patients, without inducing significant tissue damage [240,241]. Complementing these findings, our recent work using a BBB-on-a-chip microfluidic platform provided a quantitative and physiological relevant system to assess FUS-induced BBB modulation. This platform confirmed that FUS markedly enhanced trans-BBB transport, serving as a robust tool for evaluating FUS-based therapeutic strategies [242].

Beyond its role in facilitating drug delivery, FUS exerts several intrinsic therapeutic effects relevant to AD pathology. For instance, scanning ultrasound alone has been shown to remove Aβ and restore cognitive function in AD mouse model [243]. Additional studies have demonstrated that FUS mitigates AD-related pathology and improves spatial memory in mouse models and patients [244]. A recent clinical study further supports the therapeutic potential of FUS, revealing that combinatory therapy using FUS and aducanumab (anti-amyloid antibody) significantly reduces Aβ aggregation in sonicated brain regions of AD patients [245].

Mechanistic investigations suggest that these therapeutic benefits may be partly mediated through glia activation: microglia and astrocytes in FUS-treated cortical regions display increases protein expression and morphological changes indicative of activation [246]. FUS also influences intercellular communication. Deng et al. reported that ultrasound stimulation of astrocytes led to a 5-fold increase in EVs release. These ultrasound-primed ADEVs were rapidly taken up by neurons, resulting in reduced internalization of Aβ_42_ and thus decreasing its neurotoxicity in vitro [247]. Moreover, FUS has been shown to enhance glymphatic transport—a glia-dependent perivascular clearance pathway—which may further facilitate the removal of pathological proteins such as Aβ [248].

Collectively, these findings position FUS as a promising modality for AD therapy, functioning both as an enabling technology for enhanced drug delivery and as a biologically active intervention that directly modulates pathology. The continued integration of FUS with EVs creates a powerful synergistic strategy: FUS provides the non-invasive, spatially precise key to unlock the BBB, while EVs serve as ideal natural nanocarriers, leveraging their innate biocompatibility and efficient BBB-penetrating ability to deliver therapeutics. This combination of precise physical access and sophisticated biological delivery is poised to significantly amplify therapeutic impact and accelerate the translation of these technologies into clinical practice.

## 9. Conclusions

EVs are increasingly recognized as critical dual agents in AD, serving as both propagators of pathology and revolutionary platforms for diagnosis and therapy. While their endogenous role in disseminating Aβ, tau, and inflammatory signals exacerbates disease, their innate biocompatibility, ability to cross the BBB, and capacity to deliver bioactive cargo highlight their potential as next-generation interventions. Therapeutically, MSC-derived EVs and engineered EVs have demonstrated the ability to promote clearance of toxicological protein, suppressing chronic neuroinflammation, and delivering targeted genetic or pharmacological cargo directly to the brain. In parallel, EV-based “liquid biopsies” are transforming the diagnostic landscape, providing minimally invasive biomarkers—such as EV-associated proteins and miRNAs—for early detection, tracking disease progression, and monitoring of therapeutic responses. Despite these advances, key challenges—including EV heterogeneity, scalable GMP manufacturing, targeted delivery efficiency, and long-term safety—must be resolved before clinical translation can be fully realized. Future directions will advance early detection platforms via EV-based microfluidic systems and pioneer the integration of EVs with FUS. This combinatorial approach will enable spatiotemporally controlled EVs delivery and amplify therapeutic efficacy. Encouragingly, early-phase clinical studies report favorable safety profiles and initial cognitive improvements, underscoring the feasibility of EV-based approaches.

In summary, EVs represent a rapidly advancing and clinically relevant frontier in AD research. As standardization and precision engineering continue to mature, EV-based diagnostics and therapeutics are poised to become integral components of future AD management, offering meaningful opportunities to modify disease courses and improve patient outcomes.

## Figures and Tables

**Table 2:** Blood-based EVs functioning as diagnostic biomarkers for AD.

EV Subtype	Source	Changes in the Components of EVs	Disease	Conclusion	References
NDEVs	Neuron	Aβ_42_, T-tau, and p-tau T181 ↑	AD aMCI	Blood exosomal Aβ_42_, T-tau, and p-tau T181 show strong concordance with CSF and offer comparable diagnostic power for AD/aMCI.	[174]
		Aβ_42_ ↑	AD aMCI	NDEVs levels of Aβ_42_ increase progressively across the AD continuum—from cognitively normal amyloid-negative individuals to amyloid-positive individuals, to those with MCI.	[175]
		Aβ_42_, T-tau, p-tau T181, p-tau S396 ↑	AD FID	NDEVs levels of p-tau T181, p-tau S396, and Aβ_1–42_ achieves 96.4% accuracy in classifying AD patients and predict disease onset 10 years prior to clinical diagnosis.	[176]
		p-tau T181,p-tau S396 ↑ NRGN, EST ↓	AD MCI	The combined profile of p-tau, Aβ_1–42_, NRGN and REST in plasma NDEVs serves as an accurate predictor for the conversion from MCI to AD dementia.	[38]
		Aβ_1–42_ ↑ NRGN, synaptophysin ↓ synaptotagmin, synaptopodin ↓	MCI	Aβ_1–42_, NRGN, synaptophysin, synapsin, and synaptopodin in NDEVs accurately differentiate patients MCI from CNC.	[177]
		p-tau T181, Aβ_42_, NRGN ↑ ProBDNF, GluR2, PSD95 ↓ GAP43, Syntaxin-1 ↓	AD	A model incorporating GluR2, proBDNF, NRGN, and GAP43 achieved an 81.3% accuracy for AD classification. Their levels correlated with cognitive scores (MMSE and COR-SOB), supporting utility for both diagnosis and progression monitoring.	[178]
		miR-373, miR-204 ↓	AD	The significant reduction in miR-204 and miR-373 in NDEVs positions them as potential biomarkers for AD.	[184]
		NMDAR2A	AD	Analysis of synaptic protein profiles in CNS-derived plasma EVs provides a robust, liquid biopsy biomarker for synaptic dysfunction on AD	[185]
		GAP43, neurogranin, SNAP25, synaptotagmin1 ↓	AD	NDEV proteins levels (GAP43) correlate with CSF, serving as effective biomarkers and enabling the prediction of AD onset 5 to 7 years prior to the emergence of cognitive impairment.	[186]
		Aβ_1–42_ ↑	AD MCI	The combination of Aβ_1–42_ levels in NDEVs accurately predicts conversion from MCI to AD dementia.	[187]
		miR-9-5p, miR-106b-5p, miR-125b-5p ↑	AD MCI MCI-AD	MiR-106b-5p is significantly overexpressed in the AD group and shows perfect accuracy in distinguish AD from cognitive normal (CN) group.	[188]
		Aβ42/40, Tau, P-tau-T181, Aβ42, miR-29c-3p ↑	SCD aMCI AD VaD	The level of miR-29c-3p in NCAM/amphiphysin 1 dual-labeled exosomes (NDEVs) of patients with subjective cognitive decline (SCD) is higher than that in healthy controls and the vascular dementia (VaD) group, and it demonstrates the best performance in diagnosing SCD, holding potential advantages for early diagnosis. The biomarkers such as Aβ42 in single-labeled exosomes have diagnostic value for aMCI and AD, but limited diagnostic value for SCD.	[189]
		let-7e-5p, miR-96-5p, miR-484 ↑ miR-99b-5p, miR-100-5p, miR-30e-5p ↓ miR-378i, miR-145-5p, miR-378c, miR-451a ↓	AD	Compared with the HC, plasma levels of sNDEV let-7e-5p, miR-96-5p, and miR-484 were significantly increased in AD group, while levels of miR-99b-5p, miR-100-5p, miR-30e-5p et al. were significantly decreased. Let-7e expressed in NDEVs could serve as a potential biomarker for AD diagnosis (AUC value of 0.9214).	[190]
ADEVs	Astrocyte	BACE-1, g-secretase, soluble Aβ_42_, sAPPβ↓ sAPPa, GDNF, P-tau T181, p-tau S396 ↑	AD FID	The levels of BACE-1 and aAPPβ in ADEVs can effectively distinguish AD patients from healthy controls, with AUC values of 0.78 and 0.83, respectively.	[65]
		C1q, C4b, factor D, fragment ↑ Bb, C5b, C3b, C5b-C9 ↑ CD46, CD59, type 1 complement receptor ↓	MCI	The changes in complement protein levels in ADEVs not only distinguish AD patients from the control group but also demonstrate high diagnostic sensitivity in identifying MCI patients at high risk of converting to AD.	[183]
		miR-29a-5p, miR-132-5p, miR-107 ↑	AD MCI MCI-AD	The expression of miR-107 shows an increasing trend among the three patient groups and can perfectly predict the incidence of AD dementia (AUC = 1.000).	[188]
MDEVs	Microglia	miR-132-5p, miR-106b-5p ↑ miR-29a-5p, miR-125b-5p ↓	AD MCI MCI-AD	The levels of miR-29a-5p and miR-106-5p is significantly reduced across all impairment groups (AUC = 0.925). MiR-132-5p and miR-125b-5p together can perfectly predict AD (AUC = 1.000).	[188]
ODEVs	Oligodendrocytes	miR-29a-5p, miR-107, miR-135b-5p ↑	AD MCI MCI-AD	miR-29a-5p, miR-107, and miR-135b-5p were significantly overexpressed in the AD group. Mir-29a-5p showed AUC = 1.000 in predicting AD incidence.	[188]

## Data Availability

No new data were created or analyzed in this study. Data sharing is not applicable to this article.

## Abbreviations

The following abbreviations are used in this manuscript:

AD	Alzheimer’s disease
Aβ	amyloid-β
APP	amyloid precursor protein
ADEVs	Astrocyte-derived extracellular vesicles
ALS	amyotrophic lateral sclerosis
ATN	amyloid beta (A), tau (T) and neurodegeneration (N)
ahaMSCs-Exos	administered allogeneic adipose-derived MSC-EVs
BD-tau	brain-derived tau
BDENs	broccoli-derived exosomes-like nanoparticles
BACE-1	β-secretase
CNS	central nervous system
CNP	2′,3′-cyclic nucleotide 3′-phosphodiesterase
CSF	cerebrospinal fluid
CNC	cognitively normal controls
DAMPs	damage-associated molecular patterns
Exo-cur	EVs derived from curcumin-primed macrophages
EVs	extracellular vesicles
EAAT1	excitatory amino acid transporter 1
FRα	folate-receptor α
FUS	focused ultrasound
GFAP	glial fibrillary acidic protein
GSK-3β	glycogen synthase kinase-3β
GMP	good manufacturing practice
GAL-3	galectin
hUCB-MSCs	human umbilical cord blood-derived mesenchymal stem cells
hUC-MSCs-EVs	exosomes from human umbilical cord mesenchymal stem cells
hiPSC-NSCs	human induced pluripotent stem cells
HHcy	hyperhomocysteinemia
Hcy	homocysteine
HTL	hcy-thiolactone
HDS-R	Hasegawa revised dementia scale
IDE	insulin-degrading enzyme
ILVs	intraluminal vesicles
IL-4	interleukin-4
L1CAM	L1 cell adhesion molecule
MSC	mesenchymal stem cell
MVBs	multivesicular bodies
MDEVs	microglia-derived extracellular vesicles
MHC	major histocompatibility complex
MBP	myelin basic protein
MSC-EVs	mesenchymal stem cell-derived extracellular vesicles
mtDNA	mitochondrial DNA
MOG	myelin oligodendrocyte glycoprotein
MSCs	mesenchymal stem cells
MAP1b	microtubule-associated protein 1b
MExo-Gem	mannose-modified exosomes loaded with gemfibrozil
MSC-RVG-Exo	MSC-derived exosomes engineered with the brain-targeting RVG peptide
MAPLEX	a novel photoinducible exosome system
MRI	magnetic resonance imaging
miRNAs	microRNAs
MCI	mild cognitive impairment
MCIC	patients with MCI who converted to AD dementia
MCIS	stable MCI
NDEVs	neuron-derived extracellular vesicles
NEP	neprilysin
NIA-AA	National Institute on aging-Alzheimer’s association
NRGN	neurogranin
NFTs	neurofibrillary tangles
NTPDases	nucleoside triphosphate diphosphohydrolases
ODEVs	oligodendrocyte-derived extracellular vesicles
PLP	proteolipid protein
p-tau	phosphorylated tau
PET	positron emission tomography
ROS	reactive oxygen species
RVG	rabies viral glycoprotein
REST	repressor element 1-silencing transcriptional
sAPPβ	soluble amyloid precursor protein β
SVZ	subventricular zone
SCD	subjective cognitive decline
VaD	vascular dementia
5-MTHF	5-Methyltetrahydrofolic acid
5-HT	serotonin
